# Proposed roadmap to stepwise integration of genetics in family medicine and clinical research

**DOI:** 10.1186/2001-1326-2-5

**Published:** 2013-02-16

**Authors:** Elisa JF Houwink, Annet W Sollie, Mattijs E Numans, Martina C Cornel

**Affiliations:** 1 Department of Clinical Genetics, Section Community Genetics, EMGO Institute for Health and Care Research, VU University Medical Center, Amsterdam, The Netherlands; 2 School for Public Health and Primary Care, Department of General Practice, Maastricht University, Maastricht, The Netherlands; 3 Julius Center for Health Sciences and Primary Care, University Medical Center Utrecht, Utrecht, The Netherlands; 4 Department of General Practice & Elderly Care, VU Medical Centre Amsterdam, Amsterdam, The Netherlands; 5 Department of Clinical Genetics, Section Community Genetics, EMGO Institute for Health and Care Research, VU University Medical Center, BS7 D247, P.O. Box 7057, 1007MB, Amsterdam, The Netherlands

**Keywords:** Genetics registration, Family history, Electronic patient record, General practice/family medicine, Clinical research

## Abstract

We propose A step-by-step roadmap to integrate genetics in the Electronic Patient Record in Family Medicine and clinical research. This could make urgent operationalization of readily available genetic knowledge feasible in clinical research and consequently improved medical care.

Improving genomic literacy by training and education is needed first. The second step is the improvement of the possibilities to register the family history in such a way that queries can identify patients at risk. Adding codes to the ICPC chapters “A21 Personal/family history of malignancy” and “A99 Disease carrier not described further” is proposed. Multidisciplinary guidelines for referral must be unambiguous. Electronical patient records need possibilities to add (new) family history information, including links between individuals who are family members. Automatic alerts should help general practitioners to recognize patients at risk who satisfy referral criteria. We present a familial breast cancer case with a BRCA1 mutation as an example.

## Background

**Figure 1:**
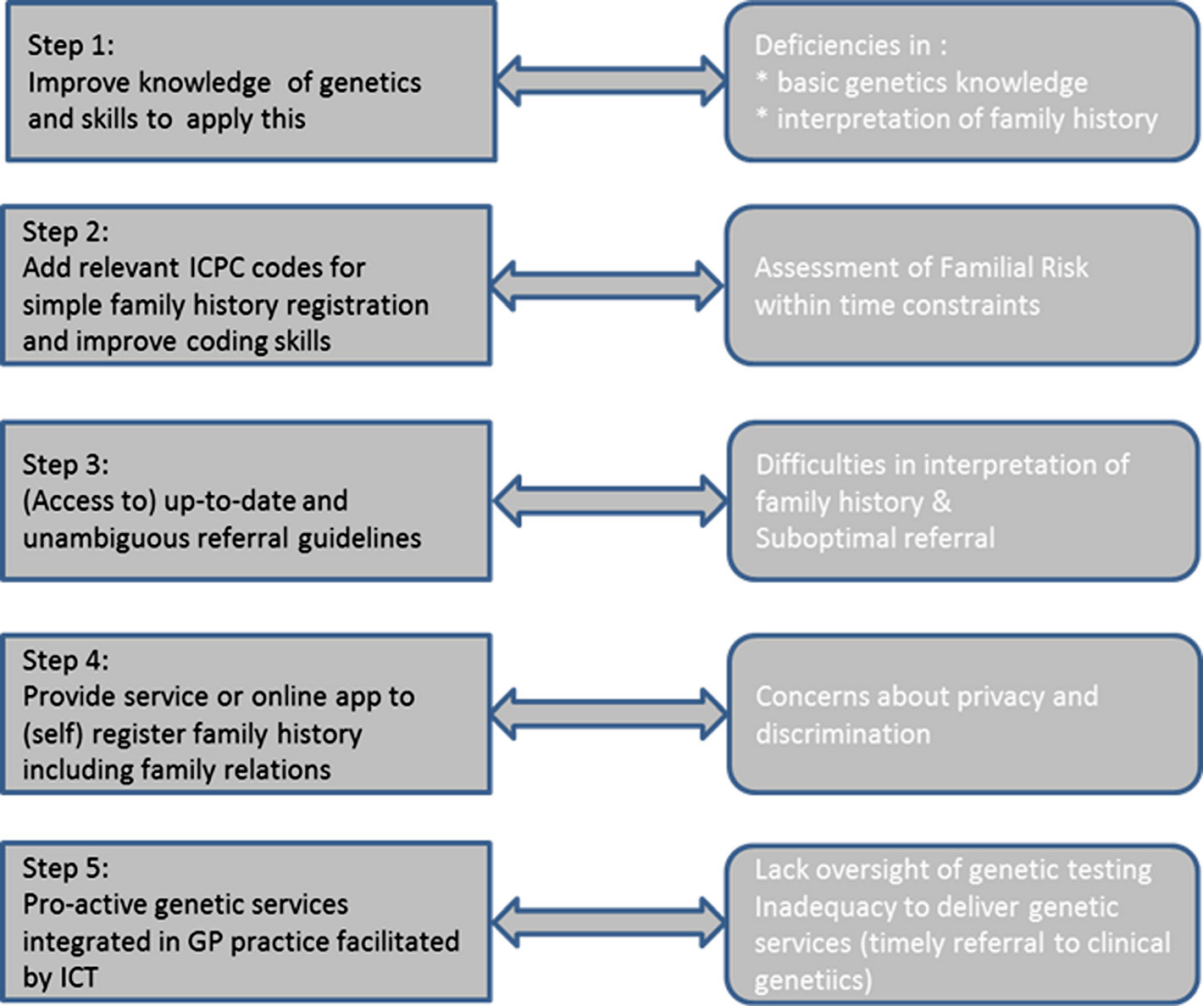
Proposed roadmap to stepwise integration of genetics in the family medicine.

Public health benefits of advancements in understanding the human genome are still to be realized for common chronic diseases such as cardiovascular disease, diabetes mellitus, and cancer [1]. International attempts to integrate and operationalize such knowledge into clinical practice are in the early stages, and as a result, many questions surround the current state of this translation [1-3]. Most physicians lack genetic knowledge and skills that might be relevant for decision support in daily practice [4]. Family history taking and family tree drawing need to be introduced. Oversight of clinical utility of genetic testing should be supported by eHealth facilities to bypass unfamiliarity with facts on genetic testing. Shortcomings in registration systems and inadequate implementation of genetics in existing guidelines are reported and result in inability to register genetic information in Electronic Patient Records. Privacy and risk of discrimination cause concerns when registration is considered. Consequently, inadequacy to deliver genetic services is reported in literature [1]. We present a roadmap (Figure 1) to integrate actual genetic knowledge into the Electronic Patient Record and into clinical research in Family medicine, which would enable urgent operationalization of readily available knowledge feasible in daily genetic medical care.

### Evidence for necessary change

The clinical relevance of integrating genetics in clinical practice was demonstrated for several familial diseases such as colorectal cancer and breast cancer. Dove-Edwin et al. calculated mortality risk reduction up to 80% by identifying and subsequently screening individuals with an increased familial colorectal cancer (CRC) risk [5]. Cancer risk management options through genetic testing for BRCA mutations and subsequent options for preventive surgery after testing positive can empower women and can also reduce morbidity and mortality [6]. Currently, a large number of patients in whom screening would be beneficial, are out of sight or being missed by their physicians [7,8].

### Barriers to change

Scheuner et al. identified deficiencies in primary care workers’ basic genetic knowledge and ability to interpret familial patterns [1]. This is in line with our prioritised educational topics, including knowledge of basic genetic principles, the most common genetic disorders and family history communication skills [9]. Taylor and Edwards stated primary care should be encouraged to invest more time and energy in questioning and registering family history data [10]. However, they also stressed identified barriers such as time constraints that should be encountered. They identified the need to develop strategies to overcome difficulties preventing general practitioners (GPs) from routinely obtaining family history information as well as strategies to support accurate record keeping in the electronic medical record (EMR) [10].

Another identified barrier is the presence of ambiguous referral guidelines to clinical genetics and other medical specialists for patients with a possible high risk at familial disease, such as cancer [9]. Computerised decision support might be helpful in familial risk assessment for common cancers (e.g. breast, ovarian and colon cancers) and would render timely genetic risk assessments and consequently support referrals more consistent with guidelines. These results support the implementation of genetics education aimed at enhancing effective referral indications and options.

### A roadmap for translation

In order to be able to truly turn useful genetic discoveries from the laboratory bench to daily clinical practice, a roadmap is crucial to make urgent translation feasible. First, advances in the genomic literacy of health care providers are indispensable. Secondly, innovative and practical ICT tools to apply these newly acquired knowledge and skills are needed, such as registration of family history and registry alerts supporting this.

We propose a step-by-step roadmap (Figure 1) to effectively integrate genetics in daily family medicine to its full potential:

1. Improve basic knowledge of genetics in clinicians and develop skills and attitude to obtain and interpret a family history through effective education;

For example, training on oncogenetics for GPs was recently developed and evaluated in collaboration with The Dutch College of Family Physicians. Also, a website on genetics targeted to GPs was developed to easily obtain information on, amongst other topics, genetic diseases, referral guidelines and family history taking (huisartsengenetica.nl, translated “GP and genetics”). Oncogenetic knowledge, skills and attitude were effectively transmitted through an accredited online and live interactive training and could internationally serve as an example for other common topics (i.e. reproductive medicine, familial coronary heart disease and diabetes) and possibly other medical specialties provided that they are *translated* to its medical systems.

2. Add relevant International Classification of Primary Care (ICPC) codes and other coding strategies for simple registry of family history and develop and support coding skills;

In order to identify and track persons and/or families at risk for hereditary diseases adequate coding is a starting point. We propose to add a number of codes for simple registration of family history. This will enable and support adequate case-finding and decision strategies [8].

### Proposal for adding codes to ICPC-2 list in case of oncogenetics

We propose to add a number of codes in order to enable simple but structured registry of a family history. In ICPC-2, which is the most frequently used coding system for GPs in Western countries, these codes should be included in Chapter A (General and Unspecified), under A21 “Risk factor for malignancy”. ICPC-2 was developed by the WHO and classifies patient data and clinical activity in the domains of General/Family Practice and primary care, taking into account the frequency distribution of problems seen in these domains. It allows classification of the patient’s reason for encounter (RFE), the problems/diagnosis managed, interventions, and the ordering of these data in an episode of care structure. ICPC-2 has a biaxial structure and consists of 17 chapters, each divided into 7 components (comp.) dealing with symptoms and complaints (comp. 1), diagnostic, screening and preventive procedures (comp. 2), medication, treatment and procedures (comp. 3), test results (comp. 4), administrative (comp. 5), referrals and other reasons for encounter (comp. 6) and diseases (comp. 7). (see http://www.who.int/classifications/icd/adaptations/icpc2/en/index.html).

Mapping is available between ICPC and ICD-10, which was also developed by the WHO for broad application in healthcare registries. The codes suggested below for breast cancer should suit other coding systems such as SNOMED as well and should also be added for other cancer types.

A21 Personal/family history of malignancy (Existing code)

A21.1 One or more 1st degree family member(s) with breast cancer

A21.2 One or more 2^nd^ degree family member(s) with breast cancer

A21.3 One or more family member(s) with bilateral or multifocal breast cancer

A21.4 Breast cancer in the family in one or more men

A99 Disease carrier not described further (Existing code)

A99.1 BRCA-1 mutation carrier

A99.2 BRCA-2 mutation carrier

A99.3 TP53 mutation carrier

A99.99 Carrier of mutation in other specified gene

3. Improve access to up-to-date and unambiguous referral guidelines;

For example, in the Netherlands multiple referral guidelines for hereditary cancers were developed independently (Oncoline, Foundation for detection of hereditary tumors (In Dutch STOET), clinical genetics centres in University hospitals and The Dutch College of Family physicians (NHG)). Limited usable information however is available for General Practitioners, i.e. only for Diagnostics of Breast Cancer and Rectal Bleeding. The guidelines are heterogeneous and difficult to interpret We propose to improve this by agreeing on national multi-disciplinary referral guidelines and provide synchronised online access to up-to-date and easy to interpret versions.

4. Provide service or online apps to (self) register family history including family relations, that can be coupled with routine healthcare registries and the EMR used in primary care too; The best way to re-use and expand previously recorded family history information and to view this history from the perspective of a different family member is by recording parent–child relations and diagnoses with the correct family member. This would require functionality to be added to the EMR. In order to overcome privacy issues an online app or website to register family history is recommended (for example: myfamilyhistory.com or familyhealthware.com).

5. Pro-active genetic services integrated in clinical practice facilitated by ICT (for example family history registry and registry alerts);

For example, the GP or nurse practitioners should be able to (periodically) register or consult family history information directly into the EMR. Accurate and up-to-date treatment and referral guidelines and subsequent automatic alerts should pop up when certain combinations of symptoms and familial risk factors indicate referral to a clinical geneticist or other medical specialist.

Illustration of the proposed roadmap with a familial breast cancer case in clinical research and family medicine:

Step 1: Patient name: Angela B., Female, age 35.

Angela lives in the city with her husband and two daughters aged 13 and 10. She works as a hair dresser, has been happily married for a decade and the family just bought a new home in the suburbs. She consults the GP on a busy Monday morning with the following complaints: Lump in left breast which she noticed during the weekend. The 4 cm irregular swelling is not painful but rather sensitive. The skin on the swelling is a little red and dimpled. Angela has no medical history, but since you followed the oncogenetic training for GPs a few weeks ago you are aware of the possible familial risks of breast cancer and decide to take her family history. Angela’s mother died of breast cancer when she was only 50 years of age 10 years ago. Her mother’s father had an unknown cancer and died at age 55. Angela tells you, when you further ask her for her family history, her sister had bilateral breast cancer at age 30 and died of ovarian cancer at age 33, two years ago. Her two other and younger sisters seem healthy.

On father’s side of the family no one has been diagnosed with cancer yet.

Step 2: If proposed codes would be added the following could be registered:

Two first-degree family members with breast cancer at an early age: mother (died at age 50) and sister (age 30, died 33, bilateral breast cancer). A21.1 and A21.3

One first-degree family member with ovarian cancer at an early age (sister age 30, died age 33).

Step 3: You are alarmed by the family history and the medical complaints of Angela. After checking the referral guidelines for cancer online, you talk with Angela about referral to the closest hospital as soon as possible for further diagnostics and possibly necessary surgical treatment. You also inform her of the chance that she might be a carrier of a DNA mutation which could be further analysed by a clinical geneticist. You promise to call the clinical geneticist and discuss the problem. The clinical geneticist agrees Angela needs further genetic DNA testing based on this positive family history and will invite her this week to quickly start DNA testing, which may inform further treatment. You call Angela afterwards and she is grateful for taking her case so seriously.

Step 4: Angela is alarmed by the fact that her positive family history for breast and ovary cancer could mean an added risk to her and her daughters to develop breast or ovarian cancer and decides to use the online tool to easily register her family history together with her family members during the upcoming family reunion. Although it was a little awkward at first to ask her family members for their medical history, they agreed to do so anonymously online and repeat this every 5 years. Angela shows her family tree online to her GP who registers relevant information in his EPD and uses this information to build a pdf with only initials and years of birth of family members and adds this to her record. Not only is she now able to take her family history to her GP, the other family members who used the online tool are also able to do so. The whole family is enabled to operationalize their family history through a snowball effect.

Step 5: Five years later Angela’s daughter Stephany, then aged 18, visits the GP with gynaecological problems. She feels a painful swelling. She started to study law in a different city and her new GP uploaded her medical and family history into his EPD. The EPD has alarmed Stephany’s new GP with a pop-up that Stephany is carrier of a *BRCA2* mutation since the clinical geneticist not only diagnosed Angela with a mutation, but unfortunately also her two daughters. Angela’s daughter is frequently checked with a physical and MRI by a surgeon familiar with familial breast- and ovarian cancer who follows the national guidelines for familial cancer. Now that she has these complaints you decide to call the surgeon and after careful deliberation you refer her the same day to the clinic for further diagnostics. Fortunately, no abnormalities are found through the gynaecological and vaginal ultrasound examination.

### Extending translational genetic competences

We offered our conceptual framework for stepwise integration of genetics into family medicine and clinical research by adding codes to the ICPC-2 list and took oncogenetics as an example. Of course this list could be further improved by adding codes in case of other diseases commonly seen in family medicine such as diabetes, cardiovascular diseases and monogenic subtypes (Maturity Onset Diabetes of the Young (MODY), *BRCA 1/2*, familial hypercholesterolemia (FH) and long QT syndrome) in particular, are expected to come increasingly to the forefront in primary care. Translational health education research is our guiding principle to improve our translational efforts and ultimately improve (genetic) medical care. Engaging colleagues in health education, clinical and biomedical research and medicine in collaborations will enhance our collective ability to move research from the “data generated from research projects” phase to the “changes in practice and policy” phase, which will then bring us full circle to finally translate genetics in to primary care. As advances both in genetic discoveries and health education research evolve, it will generate interdisciplinary collaborative endeavors within the broader scope of public health and medicine. Impact of these advances will only become manifest in better decision-making, better advocacy, better health policy and finally improved health if GPs could play a key role in translating potentially life-saving advancements in genetic technologies to patient care. If GPs are to make an effective contribution in this area, not only their competencies need to be upgraded by offering suitable and effective genetics training, but performance in real practice needs to be facilitated as well by operationalizing integration of genetics in Electronic Patient Records.

## Endnote

The manuscript contains original material which is not under review elsewhere. The study on which the research is based has been submitted to appropriate ethical review. We have not submitted this report to any other journal.

## Competing interests

The authors declare no conflicting interests.

All authors have completed the Unified Competing Interest form (available on request from the corresponding author) and declare: EJFH and MCC had financial support from Netherlands Genomics Institute for the submitted work; no financial relationships with any organizations that might have an interest in the submitted work in the previous 3 years; no other relationships or activities that could appear to have influenced the submitted work.

## Authors’ contributions

Authors EJFH and AWS conceptualized the manuscript. EJFH drafted the manuscript. AWS made significant additions and revisions to the manuscript. Both authors conceptualized and designed the frameworks. All authors, EJFH, AWS, MEN and MCC read and approved the final manuscript.
